# Severe Syndrome of Inappropriate Antidiuretic Hormone Secretion (SIADH) Following the Initiation of Valbenazine for Tardive Dyskinesia: A Case Report

**DOI:** 10.7759/cureus.58493

**Published:** 2024-04-17

**Authors:** Adeniyi A Adelakun, Clara Choi, Jeffrey Brensilver

**Affiliations:** 1 Internal Medicine, The New York Medical College Graduate Medical Education Program at Saint Mary's General Hospital and Saint Clare's Health, Denville, USA; 2 Internal Medicine, Touro College of Osteopathic Medical School, New York, USA

**Keywords:** low sodium, tardive dyskinesia, valbenazine tosylate, hyponatremia seizure, severe siadh

## Abstract

Hyponatremia, a common electrolyte disorder, usually has a benign clinical course. However, patients with the syndrome of inappropriate antidiuretic hormone secretion (SIADH) can suffer unfavorable outcomes, including mortality. Atypical antipsychotics, which are among the drugs associated with SIADH, also cause tardive dyskinesia, a condition that physicians can now effectively manage with the recently approved agent - valbenazine. We herein report a case of severe hyponatremia due to SIADH in a 58-year-old man who developed hyponatremia-induced generalized seizures six weeks after valbenazine was added to his regimen to mitigate olanzapine-associated tardive dyskinesia. His electrolyte derangement and clinical course improved following prompt recognition and treatment of SIADH. The temporal association between the commencement of valbenazine and the onset of SIADH suggests a possible but previously unreported link between valbenazine and the development of SIADH. Awareness of this uncommon association is relevant to patient safety.

## Introduction

Hyponatremia due to syndrome of inappropriate antidiuretic hormone secretion (SIADH) is characterized by inappropriately concentrated urine, associated with a state of mild volume expansion with abundant excretion of sodium in the urine. Confirming the diagnosis also requires excluding other causes of hyponatremia (e.g., hypothyroidism, adrenal insufficiency, etc.) [1]. The known causes of SIADH span a plethora of medical and surgical conditions, including intracranial infection, trauma, head injury, stroke, lung cancer, and pneumonia, as well as drugs such as opiates, antiepileptics, cytotoxics, atypical antipsychotics, etc. [2]. However, SIADH has not been described in the context of valbenazine usage. Valbenazine (INGREZZA®, Neurocrine Biosciences, San Diego, California) is the only selective vesicular monoamine transporter 2 (VMAT2) inhibitor that is FDA-approved for the treatment of Tardive dyskinesia and Huntington's disease chorea [3]. Valbenazine was not identified as a cause of hyponatremia in preclinical trials.

Post-marketing surveillance and real-world case reports are critical for detecting and illuminating uncommon adverse drug reactions not recorded in the pre-clinical and clinical phases of drug development. We present a patient with chronic Schizophrenia who was treated with valbenazine for olanzapine-associated tardive dyskinesia but subsequently required intensive care unit admission for severe generalized seizures. A laboratory work-up confirmed severe hyponatremia secondary to SIADH as the underlying cause, and his condition resolved with the prompt treatment of SIADH.

## Case presentation

A 58-year-old man was brought to the emergency department (ED) after an observed seizure. He was intubated and sedated in the field by the paramedics. Further information obtained from his family members was that the patient had been acting “off'' for a few days with concomitant lethargy and anorexia before the acute seizure episode. There is no report of fever, vomiting, or diarrhea. His medical history was notable for hypertension, benign prostate hypertrophy, schizophrenia, and a new assessment of tardive dyskinesia; he had a colonoscopy one week before presentation. His medications included esomeprazole, amlodipine, atorvastatin, tamsulosin, valbenazine tosylate (Ingrezza™) 40mg 1 capsule/day, paliperidone palmitate IM injection every three months, and olanzapine 15mg 1 tablet/day. He had been on olanzapine and paliperidone for many years and began treatment with valbenazine for the treatment of tardive dyskinesia six weeks before his presentation with seizures. He had no reported allergies or adverse drug reactions, and his social history included smoking half a pack per day.

The vital signs on admission reveal a temperature of 97.8 °F, blood pressure of 141/82 mmHg, pulse of 80 beats per minute, oxygen saturation of 100% on room air, and respiratory rate of 29 cycles per minute. During the physical examination, the patient was unresponsive but spontaneously moved all limbs. He had increased muscle tone in all limbs; neurologic examination could not be completely evaluated because he was sedated. The initial laboratory studies revealed severely low serum sodium; however, serum potassium, chloride, carbon dioxide, blood urea nitrogen, creatinine, calcium, and lactate were unremarkable (Table 1).

**Table 1:** Admission basic metabolic panel and complete blood count. Laboratory values from the patient upon arrival in the ED (before the commencement of treatment of hyponatremia).

Investigation	Patient values	Reference range
Sodium	111 mmol/L	136 - 145 mmol/L
Potassium	4.3 mmol/L	3.5 - 5.3 mmol/L
Chloride	80 mmol/L	98 - 110 mmol/L
CO2	22.0 mmol/L	20.0 - 31.0 mmol/L
Anion Gap	9.0 mmol/L	6 - 12 mmol/L
BUN	9.0 mg/dl	6.0 - 24 mg/dL
pH	7.454	7.340 - 7.454
pCO2	34.1 mmHg	35 - 48.0 mmHg
pO2 (fio2: 100%)	232.7 mmHg	75 - 100.0 mmHg
HCO3	23.8 mmol/L	22.0 - 28.8 mmol/L
Lactate	1.43	0.5 - 2.2
WBC	10.70 X 10^3^/uL	4.80 - 10.80 X 10^3^/uL
RBC	4.09 X 10^6^/uL	4.32 - 5.72 X 10^6^/uL
Hemoglobin	12.5 g/dL	13.5 - 17.5 g/dL
Platelets	182 X 10^3^/uL	150 - 450 X 10^3^/uL

Random serum osmolality was low, urine osmolality was in the high normal range, and urine specific gravity was within range. The complete blood count, blood glucose, and TSH were unremarkable. The chest radiograph showed normal findings. The CT head showed no acute intracranial findings, and both, CT angiograms of the head and neck showed no hemodynamically significant stenosis. The brain non-contrast MRI was also normal. The EEG showed generalized slowing without evidence of seizures during the study. Lastly, drug toxicology screening (ethanol, amphetamine, barbiturate, benzodiazepine, cannabinoid, cocaine, opiate, and PCP) was negative.

Severe hyponatremia and reduced plasma osmolality with increased urinary osmolality and elevated urine sodium concentration in the absence of an alternative explanation for the cause of the hyponatremia supported a diagnosis of SIADH. This assessment is also consistent with the Barter and Schwartz diagnostic criteria for SIADH viz: decreased serum osmolality (<275 mOsm/kg), increased urine osmolality (>100 mOsm/kg) euvolemia, increased urine sodium (>20 mmol/L), and no other cause for hyponatremia [2].

An intravenous infusion of 100ml of 3% saline was administered, followed by 0.9% normal saline at 100 ml/hr. He was also given 2 $\mu$g parenteral desmopressin. At 8 hours post-admission, serum sodium had risen above goal; the rest of the labs, including potassium, chloride, carbon dioxide, blood urea and nitrogen, creatinine, glucose, and calcium, remained within normal range while urine sodium decreased (Table 2).

**Table 2:** Paired Osmolality and Urine Electrolytes

Urine electrolyte	Admission	At 3 hours	Reference range
Urine Sodium	48 mmol/L	21 mmol/L	28 - 272 mmol/dL
Urine Chloride	63 mmol/L	11 mmol/L	32 - 290 mmol/L
Urine Potassium	38.4 mmol/L	4.1 mmol/L	12 - 129 mmol/L
Urine Specific Gravity	1.013	-	1.005 - 1.030
Urine Osmolality, random	355 mOsmol/kg	-	50 - 1400 mOsmol/kg
Serum Osmolality	254 mOsmol/kg	-	275 - 295 mOsmol/kg

The sodium correction goal of less than 6 in the first 6 hours was exceeded; intravenous fluid was then replaced with a 5% dextrose water infusion at 100 cc/hr, which was continued for 16 hours because sodium over-correction remained above the intended target in the first 24 hours.

The serum electrolytes were monitored in the first 24 hours. Between eight hours post-admission and the end of the first 24 hours, serum sodium ranged between 123 mmol/L and 130 mmol/L. On the second day, serum sodium gradually increased to the normal limit, as shown in Table 3.

**Table 3:** Serum Sodium Trend Serum sodium values throughout admission. Reference range: 136 - 145 mmol/L.

Hours post admission	Sodium	Change in Na since admission
1st hour of admission	111	-
7 hour(s) 32 minute(s)	123	12
12 hour(s) 26 minute(s)	126	15
15 hour(s) 48 minute(s)	128	17
20 hour(s) 52 minute(s)	130	19
25 hour(s) 26 minute(s)	128	17
31 hour(s) 25 minute(s)	129	18
37 hour(s) 21 minute(s)	131	20
42 hour(s) 56 minute(s)	136	25
49 hour(s) 16 minute(s)	134	23
56 hour(s) 28 minute(s)	134	23
59 hour(s) 52 minute(s)	134	23
81 hour(s) 22 minute(s)	135	24
105 hour(s) 9 minute(s)	136	25
128 hour(s) 1 minute(s)	138	27
152 hour(s) 18 minute(s)	136	25

On the second day of admission, after a successful weaning attempt, the patient was extubated. On day 3, he passed the bedside swallow test, stepped down to the medical floor, and was discharged on day six after making remarkable gains with the physical therapist. Valbenazine was withheld from day 1 of admission.

## Discussion

Valbenazine is converted to its active metabolite [+]-$\alpha$-dihydro tetrabenazine, which also binds to human VMAT2. However, both active agents have no affinity for dopaminergic (including D2), serotonergic (including 5HT2B), adrenergic, histaminergic, or muscarinic receptors [4,5]. According to the INGREZZA package insert, the side effects of valbenazine include somnolence and prolongation of corrected QT interval in patients who are CYP2D6 poor metabolizers or others who are taking CYP2D6 OR CYP3A4 inhibitors. Valbenazine may also cause Parkinson-like symptoms. It has a potential risk to fetuses. In 2017, Valbenazine was approved by the FDA for treating Tardive dyskinesia (TD) [5].

In KINECT 3, emergent adverse events are defined as untoward events that occurred or worsened in severity after the first dose of the study drug. The study also included various safety assessments, including vital signs, physical examinations, ECG, laboratory tests, the Simpson-Angus Scale, and the Barnes Akathisia Rating Scale [6]. We observed that in KINECT 3, neither hyponatremia nor SIADH was reported as distinct adverse events in the 6-week study. However, in the same study, patients were monitored for weight gain, a potential proxy for fluid retention that could be observed in hyponatremia or SIADH. Weight gain was reported in the two treatment groups of the study (1 out of 72 (1.4%) subjects on valbenazine 40mg daily and 2 out of 79 subjects (2.5%) on valbenazine 80mg daily) but none in the placebo group [6].

Severe hyponatremia due to SIADH, presenting with seizures six weeks after the initiation of valbenazine, suggests a possible link between this medication and SIADH. The absence of alternative explanations for hyponatremia and SIADH despite a careful search (including laboratory and imaging studies) supports our suggestion. We are aware that patients with schizophrenia could present with hyponatremia secondary to psychogenic polydipsia, but increased urinary sodium concentration is not observed in that cohort of patients [7,8].

The use of atypical antipsychotics has been recognized as a possible cause of SIADH [2]. However, we believe that the temporal association between the initiation of valbenazine and the emergence of our patient’s symptoms suggests a recent trigger. This is also corroborated by the fact that he had been on olanzapine for 18 years; olanzapine was continued during the course and after admission. Also, the possibility of synergism between valbenazine and olanzapine in the emergence of SIADH remains open for mechanistic evaluation [9]. A colonoscopy in the week preceding the admission for seizure was not deemed a factor in the development of the patient’s severe complicated hyponatremia, which was characterized by inappropriately concentrated urine with abundant sodium.

Despite diligently searching the medical literature, we are still looking for a previous publication on the association between the use of valbenazine and the development of hyponatremia or SIADH. Furthermore, we communicated with Neurocrine Biosciences (the manufacturer of INGREZZA), and they confirmed that only one hyponatremia was observed during the clinical trials.

## Conclusions

Based on the clinical profile of our patient and the absence of any other cause of SIADH, we suggest that there may be a link between the use of valbenazine and the emergence of SIADH. We urge clinicians treating patients with valbenazine to consider the potential risk of severe hyponatremia due to SIADH, especially in patients who are inclined to drink copious amounts of fluids in the contexts of their primary morbidity, comorbidities, or ongoing medications.
